# Impact on Survival of Early Versus Late Initiation of Adjuvant Chemotherapy After Pancreatic Adenocarcinoma Surgery: A Target Trial Emulation

**DOI:** 10.1245/s10434-023-14497-x

**Published:** 2023-11-01

**Authors:** Jakob Kirkegård, Morten Ladekarl, Andrea Lund, Frank Mortensen

**Affiliations:** 1 HPB Section, Department of Surgery, Aarhus University Hospital, Aarhus, Denmark; 2 Department of Clinical Medicine, Aarhus University, Aarhus, Denmark; 3 Department of Oncology and Clinical Cancer Research Center, Aalborg University Hospital, Aalborg, Denmark; 4 Department of Clinical Medicine, Aalborg University, Aalborg, Denmark

**Keywords:** Pancreatic adenocarcinoma, Surgery, Adjuvant chemotherapy, Treatment delay, Survival

## Abstract

**Background:**

We examined the impact of early (0–4 weeks after discharge) versus late (> 4–8 weeks after discharge) initiation of adjuvant chemotherapy on pancreatic adenocarcinoma survival.

**Methods:**

We used Danish population-based healthcare registries to emulate a hypothetical target trial using the clone-censor-weight approach. All eligible patients were cloned with one clone assigned to ‘early initiation’ and one clone assigned to ‘late initiation’. Clones were censored when the assigned treatment was no longer compatible with the actual treatment. Informative censoring was addressed using inverse probability of censoring weighting.

**Results:**

We included 1491 patients in a hypothetical target trial, of whom 32.3% initiated chemotherapy within 0–4 weeks and 38.3% between > 4 and 8 weeks after discharge for pancreatic adenocarcinoma surgery; 206 (13.8%) initiated chemotherapy after > 8 weeks, and 232 (15.6%) did not initiate chemotherapy. Median overall survival was 30.4 and 29.9 months in late and early initiators, respectively. The absolute differences in OS, comparing late with early initiators, were 3.2% (95% confidence interval [CI] − 1.5%, 7.9%), − 0.7% (95% CI − 7.2%, 5.8%), and 3.2% (95% CI − 2.8%, 9.3%) at 1, 3, and 5 years, respectively. Late initiators had a higher increase in albumin levels as well as higher pretreatment albumin values.

**Conclusions:**

Postponement of adjuvant chemotherapy up to 8 weeks after discharge from pancreatic adenocarcinoma surgery is safe and may allow more patients to receive adjuvant therapy due to better recovery.

**Supplementary Information:**

The online version contains supplementary material available at 10.1245/s10434-023-14497-x.

Pancreatic adenocarcinoma has a poor prognosis,^1^ and even after curative-intent surgery, the 5-year survival is only around 25%.^2^ Adjuvant chemotherapy is known to improve survival considerably, with modified FOLFIRINOX, gemcitabine combined with capecitabine or nab-paclitaxel, and, in Japan, S1, providing better survival than gemcitabine monotherapy.^3–9^ However, around half of the patients do not initiate adjuvant chemotherapy, which may be due to poor performance, early tumor recurrence, and postoperative complications.^10,11^

Several observational studies have examined if the timing of adjuvant chemotherapy affects survival, most of which found no clinically meaningful difference between early and late initiation of adjuvant chemotherapy.^12–29^ However, both comparability between the studies and generalizability to other target populations are limited. There were major differences in the definitions of early and late initiation, median survival times ranged from 20 to 40 months, several studies did not specify the upper limit for allowed waiting times, and most studies used the same data source (the US-based National Cancer Database) and thus had overlapping study populations. More importantly, most studies were substantially affected by immortal time bias (by using information captured after the start of follow-up to define exposure groups at baseline) or selection bias (introduced by exclusion of non-initiators), limiting interpretability. Both of these study design-related biases are known to introduce spurious associations when estimating treatment effects.^30,31^

Target trial emulation is a novel study design that specifically addresses the potential for immortal time bias and selection bias.^32^ Using this design, we aimed to provide evidence on the impact of timing of initiation of adjuvant chemotherapy on survival after pancreatic adenocarcinoma surgery, by examination of the effect of adjuvant chemotherapy initiated within 4 weeks compared with initiation between 4 and 8 weeks after discharge for pancreatic adenocarcinoma surgery.

## Methods

### Setting and Data Sources

We linked data from nationwide healthcare registries in Denmark to identify patients undergoing surgery for pancreatic adenocarcinoma during the period 2008–2022.^33–37^ We used information from The Danish National Patient Registry,^33^ Danish Cancer Registry,^34^ Civil Registration System,^35^ Danish Anesthesia Database,^36^ Danish Pathology Registry,^37^ and the Register of Laboratory Results for Research.^38^ Detailed descriptions of the data sources are provided in eTable 1 in the electronic supplementary material.

### Study Design and Population

We specified a hypothetical target trial that would ideally be conducted to answer our research question, and then emulated this trial using observational data.^32^

#### The Target Trial

Details of the target trial and our emulation are specified in ESM eTable 2. In brief, this would be a randomized trial of previously untreated patients undergoing radical resection for pancreatic adenocarcinoma without distant metastases, eligible for postoperative chemotherapy and randomized to either early (0–4 weeks after discharge) or late (> 4–8 weeks after discharge) initiation of adjuvant chemotherapy. The primary endpoint was the difference in median overall survival (mOS), while secondary endpoints were absolute difference in 1-, 3-, and 5-year survival.

#### Emulation Using Observational Data

To emulate the target trial using observational data, we initially included all patients aged at least 18 years who were undergoing surgery for pancreatic adenocarcinoma and who were registered in the Danish National Patient Registry during the period 2008–2022 (*n* = 2580). The index date was set to the date of discharge after surgery. We excluded patients who died during the index admission (*n* = 62), had less than 5 years of continuous residency in Denmark before surgery (*n* = 9), had an unknown area of residence (*n* = 11), had received neoadjuvant treatment (*n* = 197), or had missing information on pathological T or N status (*n* = 215). Furthermore, to avoid non-positivity, we restricted the study population to patients meeting the inclusion criteria of the target trial (i.e. patients assumed to be eligible for adjuvant chemotherapy at the time of discharge). Thus, we excluded patients with a metastatic tumor on postoperative pathology or a record of tumor recurrence before the index date (*n* = 82). Tumor recurrence was defined as either (1) a biopsy with verified malignancy compatible with pancreatic adenocarcinoma in the lung, liver, or peritoneum; (2) an International Classification of Diseases (ICD) code of metastatic malignancy; or (3) receipt of *nab*-paclitaxel, as this treatment regimen is only used in a palliative setting in Denmark (ESM eTable 3). We excluded patients with a diagnosis of another solid tumor (except biliary tract or duodenal cancers, as these were likely wrongly coded pancreatic cancers, and non-melanoma skin cancer) during the year before the index date (*n* = 79). We also required that patients were considered, in performance, to initiate adjuvant chemotherapy. We approximated this information by excluding patients with a length of stay after surgery of > 4 weeks (*n* = 181), Clavien–Dindo score of IV or American Society of Anesthesiologists (ASA) score of IV (*n* = 26), body mass index (BMI) < 17, or a Nordic Multimorbidity Index (NMI)^39^ score of > 20 (combined *n* = 51). We furthermore excluded patients with latest available blood levels of bilirubin > 50 umol/L, hemoglobin < 5 mmol/L, platelets < 100 × 10^9^/L, creatinine > 100 umol/L, CA19-9 > 200 U/L, and albumin < 20 g/L (combined *n* = 176). For details, see ESM eFig. 1.

### Treatment Strategies

In the target trial, treatment would be assigned randomly when eligibility was determined. In our trial emulation, we used an 8-week grace period from the index to capture the two treatment strategies of initiation of adjuvant chemotherapy within 0–4 weeks (early initiation) or > 4–8 weeks (late initiation) following discharge after surgery. A maximum of 8 weeks was chosen to decrease the risk of including patients receiving palliative treatment for recurrences prior to the index date. We restricted data to chemotherapy codes recorded at oncological departments.

### Covariates

We included information on comorbidities, BMI, ASA score, and blood tests, in addition to information related to the index admission and postoperative complications. Missing values on BMI, ASA score, and blood samples were addressed using multiple imputation (except for CA19-9 due to a too-high proportion of missing values). For all patients, we retrieved a full medical history of diagnoses recorded up to 5 years before the index date (ESM eTable 4). To augment the assessment of comorbidities, we identified prescriptions used to treat the relevant comorbidities, restricting to a 1-year lookback period (ESM eTable 4). We constructed a composite score of the overall comorbidity burden for each patient using the NMI, a validated comorbidity index designed to predict 5-year mortality in a Danish population (ESM eTable 5).^39^ We obtained information on the length of stay for the admission related to the surgery (index admission) and Clavien–Dindo score.^40^ When information on the Clavien–Dindo score was unavailable, we used information on procedures and treatments recorded during the index admission (ESM eTable 6). We constructed a composite measure of postoperative complications defined as low (Clavien–Dindo score ≤ II) and high (Clavien–Dindo score III). Data sources of all covariates are shown in ESM eTable 1. For BMI, ASA score, and blood samples, we used the most recent measurement recorded in the period between the date of surgery and the index.

### Statistical Analyses

#### Main Analysis

We analyzed our study using the clone-censor-weight approach.^41,42^ First, each individual was duplicated in the dataset at the index date (date of discharge). One copy was then assigned to the early initiation strategy and the other copy to the late initiation strategy. Second, the copies were artificially censored over time when the observed treatment deviated from the assigned treatment. Copies assigned to the early initiation strategy were censored after 4 weeks if adjuvant chemotherapy was not initiated, and the copies assigned to the late initiation strategy were censored if adjuvant chemotherapy was initiated before 4 weeks or not initiated after 8 weeks. Follow-up ended at death, emigration (*n* < 5), after 5 years, or on 23 July 2023, whichever occurred first.

Since treatment was not randomized, potential confounding variables (age, sex, year of diagnosis, marital status, area of residence, comorbidity, BMI, ASA score, tumor stage, postoperative complications, blood samples) may have influenced the actual treatment decision, and thereby deviation from the assigned treatment strategy. Covariates were identified using a practice- and literature-informed directed acyclic graph (ESM eFig. 2). Therefore, in our third step, we used inverse probability of censoring weighting to account for the potential selection bias caused by informative censoring.^32^ Treatment-specific weights were derived using pooled logistic regression models as outlined in ESM eFig. 3. Weights were truncated at the 1st and 99th percentile to reduce the impact of extreme weights.^43^ Throughout the grace period, comorbidity, BMI, ASA score, and blood samples were updated daily to guard against time-varying confounding (ESM eTable 7). To assess covariate balance, we calculated the standardized mean differences of potential confounders between the two strategies before and after weighting at the end of the grace period. A covariate with an absolute standardized mean difference of < 0.10 was considered sufficiently balanced.^43^ We report the mOS and overall survival at 1, 3, and 5 years after diagnosis, calculated using the Kaplan–Meier estimator. The treatment effects were contrasted using absolute differences. All estimates are presented with standard error-derived 95% confidence intervals (CIs), calculated using bootstrapping with 200 repetitions. We report estimates for all patients and stratified by N stage, which may introduce effect modification.^44^ Statistical analyses were conducted using Stata 18 (StataCorp LLC, College Station, TX, USA).

#### Supplemental and Sensitivity Analyses

To further explore and quantify the impact of timing of adjuvant chemotherapy on survival in pancreatic adenocarcinoma patients, we conducted a supplemental analysis on the non-cloned cohort using a Cox proportional hazards regression model. To investigate the impact of exposure dichotomization,^45^ we started follow-up on the date of initiation of adjuvant chemotherapy, and modeled the time from discharge to start of adjuvant chemotherapy as a restricted cubic spline with three knots. Outcome was overall survival. All covariates were modeled similar to the target trial emulation, and this analysis was also stratified by N stage. As a marker of postsurgical recovery,^46^ we also examined changes in albumin levels from the date of discharge to the date of treatment initiation. Paired data were analyzed using a Wilcoxon signed-rank test, and unpaired data were analyzed using a Wilcoxon rank-sum test. To examine the robustness of our findings, we conducted two sensitivity analyses designed to address potential positivity violations. First, we did not apply any restrictions on eligibility criteria, and second, we excluded patients with an ASA score of III to test a less and more restrictive approach, respectively.

### Ethical Considerations

This study was approved by the Danish Data Protection Agency and the Danish Health Data Authority. Ethical approval is not required for registry-based studies in Denmark.

## Results

### Descriptive Characteristics

We included 1491 patients who were discharged alive within 4 weeks after surgery for pancreatic adenocarcinoma (Table 1). The median age was 69 years (interquartile range [IQR] 62–74 years), and 51.7% were men. Of the 1491 patients, 482 (32.3%) initiated adjuvant chemotherapy within 0–4 weeks and 571 (38.3%) initiated treatment between > 4 and 8 weeks after discharge for pancreatic adenocarcinoma surgery; 206 (13.8%) initiated chemotherapy after > 8 weeks, and 232 (15.6%) did not initiate adjuvant chemotherapy. Characteristics on pathology and surgical details are presented in Table 2. There were no differences according to type of chemotherapy (gemcitabine monotherapy, gemcitabine combinations, or mFOLFIRINOX) received between early and late initiators. All characteristics according to receipt or non-receipt of adjuvant chemotherapy are presented in ESM eTables 8 and 9.

**Table 1:** Descriptive characteristics of the study population

	*N* (%)
Total	1491 (100)
Age, years [median (IQR)]	69 (62–74)
Age group, years	
<60	318 (21.3)
61–70	557 (37.4)
> 70	616 (41.3)
Sex	
Men	771 (51.7)
Women	720 (48.3)
Marital status	
Married/registered partner	684 (45.9)
Unmarried/divorced/widowed	807 (54.1)
Area of residence	
Urban municipality	982 (65.9)
Rural municipality	509 (34.1)
Calendar period of diagnosis	
2008–2014	446 (29.9)
2015–2018	477 (32.0)
2019–2022	568 (38.1)
Alcohol consumption	
None	160 (10.7)
1–14 units per week	241 (16.2)
15–21 units per week	10 (0.7)
> 21 units per week	14 (0.9)
Unknown	1066 (71.5)
Tobacco smoking	
Non-smoker	363 (24.3)
Current smoker	143 (9.6)
Former smoker	57 (3.8)
Unknown	928 (62.2)
Nordic Multimorbidity Index, mean (SD)	3.2 (4.6)
Body mass index, mean (SD); imputed: 55.3%	24.8 (3.1)
ASA score (imputed: 57.9%)	
I	22 (1.5)
II	888 (59.6)
III	581 (39.0)
Comorbidity	
Stroke or other cerebrovascular disease	40 (2.7)
Cardiac disease	258 (17.3)
Hypertension	807 (54.1)
Chronic lung disease	243 (16.3)
Diabetes	444 (29.8)
Chronic liver disease	19 (1.3)
Kidney disease	12 (0.8)
Alcohol-related disease	50 (3.4)
Smoking-related disease	188 (12.6)
Psychiatric disease	74 (5.0)
Blood tests (median (IQR))	
CRP, mg/L (imputed: 18.0%)	29 (14–59)
Hgb, mmol/L (imputed: 17.8%)	6 (6–7)
Leukocytes, 10^9^/L (imputed: 17.3%)	10 (8–12)
Platelets, 10^9^/L (imputed: 21.5%)	393 (277–504)
Albumin, g/L (imputed: 37.4%)	27 (25–30)
Bilirubin, umol/L (imputed: 19.7%)	12 (7–21)
CA19-9, U/L (missing: 98.3%)	57 (28–99)
Creatinine, umol/L (imputed: 17.2%)	58 (49–69)

*IQR* interquartile range, *ASA* American Society of Anesthesiologists, *CRP* C-reactive protein, *Hgb* hemoglobin

**Table 2:** Tumor and surgery characteristics of the study population

	*N* (%)
Total	1491 (100)
Tumor location	
Head	946 (63.4)
Body	104 (7.0)
Tail	109 (7.3)
Multiple	91 (6.1)
Unknown	241 (16.2)
Tumor size, mm [median (IQR)]	30 (23–37)
pT stage	
T1	126 (8.5)
T2	461 (30.9)
T3	851 (57.1)
T4	53 (3.6)
pN stage	
N0	442 (29.6)
N+	1049 (70.4)
AJCC stage	
I	225 (15.1)
II	986 (66.1)
III	280 (18.8)
Tumor differentiation	
Poor	162 (10.9)
Moderate	269 (18.0)
High	48 (3.2)
Unknown	1012 (67.9)
Neural invasion	
No	53 (3.6)
Yes	405 (27.2)
Unknown	1033 (69.3)
Vascular invasion	
No	131 (8.8)
Yes	327 (21.9)
Unknown	1033 (69.3)
Microradical resection	
No	802 (53.8)
Yes	136 (9.1)
Unknown	553 (37.1)
Type of surgery	
Pancreatoduodenectomy	186 (12.5)
Distal pancreatectomy	1146 (76.9)
Total pancreatectomy	159 (10.7)
Length of stay, days [median (IQR)]	10 (8–15)
Complication score	
Low (Clavien–Dindo 0–II)	1258 (84.4)
High (Clavien–Dindo III)	233 (15.6)

*IQR* interquartile range, *AJCC* American Joint Committee on Cancer

### Survival Estimates

**Fig. 1:**
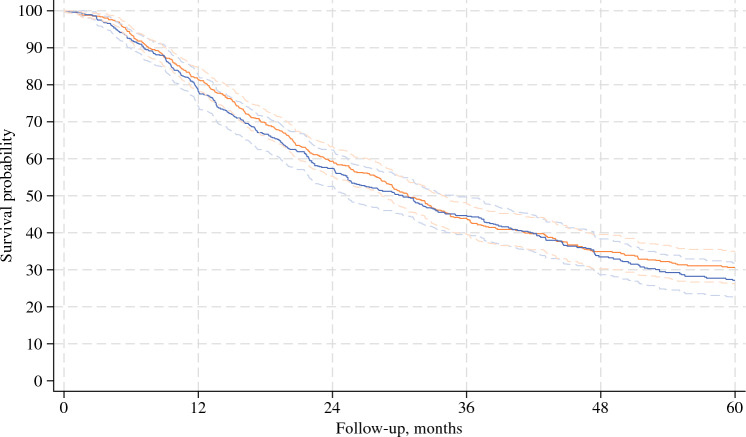
Overall survival since date of discharge for pancreatic adenocarcinoma surgery, comparing the early (blue) and late (orange) initiation strategies. Weighted estimates. Lines are shown with 95% confidence intervals

**Fig. 2:**
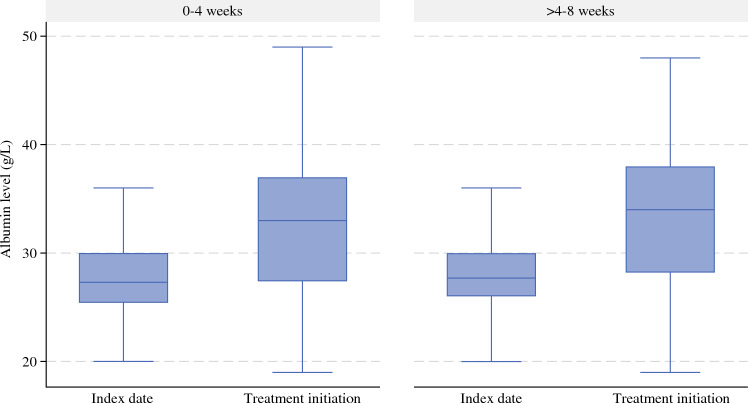
Median albumin levels among early and late initiators at the index date and the date of treatment initiation. 0–4 weeks: comparison of the index date and treatment initiation: *p* < 0.05; > 4–8 weeks: comparison of the index date and treatment initiation: *p* < 0.05. Outliers not shown due to the protection of individual-level data

After weighting, all covariates were sufficiently balanced at the end of the grace period (ESM eFig. 4). mOS was 29.9 months (IQR 13.4–not reached [NR]) in early initiators and 30.4 months (IQR 15.1–NR) in late initiators, corresponding to an absolute difference of 0.5 months (95% CI − 5.1, 6.0) [Fig. 1]. The absolute difference in overall survival, comparing late initiators with early initiators, was 3.2% (95% CI − 1.5%, 7.9%), − 0.7% (95% CI − 7.2%, 5.8%), and 3.2% (95% CI − 2.8%, 9.3%) at 1, 3, and 5 years, respectively (Table 3). There was a slight tendency towards better survival in late initiators compared with early initiators in N0 patients (ESM eFig. 5), but this is likely explained by insufficient covariate balance and thus residual selection bias. In both early and late initiators, we observed an increase in blood levels of albumin at treatment initiation from that at the index date (Fig. 2). The absolute increase in albumin was higher in late initiators compared with early initiators (absolute increase 5.8 vs. 4.8 g/L; *p* = 0.0015).

**Table 3:** Survival estimates at 1, 3, and 5 years after discharge according to treatment strategy in the target trial emulation

	Survival, % (95% CI)		
	1 year	3 years	5 years
All patients			
Late initiators	81.4 (78.4, 84.4)	43.9 (39.7, 48.2)	30.4 (26.0, 34.8)
Early initiators	78.2 (74.1, 82.2)	44.6 (39.5, 49.7)	27.2 (22.5, 31.8)
Difference	3.2 (− 1.5, 7.9)	− 0.7 (− 7.2, 5.8)	3.2 (− 2.8, 9.3)
N+ patients			
Late initiators	77.1 (73.3, 80.9)	33.5 (28.6, 38.5)	19.6 (15.3, 23.9)
Early initiators	75.6 (70.9, 80.2)	37.9 (32.1, 43.7)	17.8 (12.6, 22.9)
Difference	1.5 (− 4.6, 7.6)	− 4.4 (− 11.8, 2.9)	1.8 (− 5.0, 8.6)
N0 patients			
Late initiators	90.9 (86.9, 95.0)	66.6 (58.5, 74.8)	53.0 (43.0, 63.0)
Early initiators	85.3 (78.3, 92.3)	60.9 (51.2, 70.6)	48.1 (36.5, 59.6)
Difference	5.6 (− 2.1, 13.4)	5.7 (− 7.2, 18.7)	5.0 (− 11.3, 21.3)

*CI* confidence interval

### Supplemental and Sensitivity Analyses

**Fig. 3:**
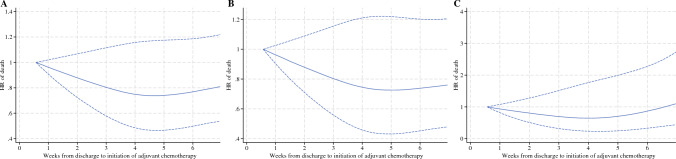
HR of death (with 95% CI) according to the time to initiation of adjuvant chemotherapy. Start of follow-up on the date of initiation of adjuvant chemotherapy for (**A**) All patients; (**B**) patients with N+ disease; and (**C**) Patients with N0 disease. *HR* hazard ratio, *CI* confidence interval

When we started follow-up on the date of initiation of chemotherapy, we observed a tendency towards improved prognosis with longer time to initiation of adjuvant chemotherapy in patients with N+ disease but not N0 disease (Fig. 3). Neither of the sensitivity analyses had any major impact on our estimates (ESM eTable 10).

## Discussion

We used target trial emulation, a rigorous framework designed to mitigate selection bias and immortal time bias, to estimate the effect of early (0–4 weeks) versus late (> 4–8 weeks) initiation of adjuvant chemotherapy on survival in patients undergoing curative-intent surgery for pancreatic adenocarcinoma. Overall, late and early initiators had similar survival.

The literature on timing of adjuvant chemotherapy after pancreatic adenocarcinoma surgery is contradictory. Most prior research found no or limited evidence of different prognosis according to timing of treatment initiation, regardless of the definitions of early or late initiation.^13,15,16,18,20,22–24,29^ Few studies found that early initiation (definition ranging from 5 to 10 weeks) was associated with an improved survival,^17,21,26–28^ whereas one study found that initiation after approximately 5 weeks conferred a survival benefit.^25^ In most studies, the magnitude of the differences was small and of limited clinical relevance; however, the studies were prone to immortal time bias and selection bias. Immortal time bias can be introduced when information on future treatments are used to create exposure groups at baseline.^30,31^ For example, when examining the impact of initiation of adjuvant chemotherapy before or after 8 weeks, patients in the > 8 weeks group will have 8 weeks of guaranteed survival time when this approach is used. If the patient had died or initiated treatment before 8 weeks, they would not be in the > 8-weeks group. Thus, immortal time bias can lead to inflated and spurious effect estimates. Selection bias can be introduced by exclusion of patients dying before treatment initiation, particularly in highly fatal malignancies such as pancreatic adenocarcinoma. It is not known which treatment these patients would have been allocated to, and it is likely to be different from that of patients who actually started adjuvant chemotherapy. Thus, inference from those in the study to the entire population is not straightforward. This may be prevalent in several papers, as suggested by the survival curves separating shortly after start of follow-up.^19,47^ Early separation of the survival curves are suggestive of selection bias, as any treatment effect would require some time to materialize.

Prior research used the date of surgery as the index date, whereas we used the date of discharge. This was chosen as we used a target trial emulation. In order to mitigate immortal time bias with this approach, eligibility and time zero (index date) should be aligned. Thus, at the date of surgery, in contrast to the date of discharge, it is not possible to determine eligibility for adjuvant chemotherapy, because postoperative complications may occur, rendering the patient ineligible for adjuvant chemotherapy. Our findings are therefore not entirely comparable with the prior literature. However, median length of stay was limited to 10 days, and we excluded patients with admission after surgery of more than 4 weeks to avoid non-positivity by inclusion of patients, who would never be eligible for chemotherapy due to poor postoperative health status.

To mitigate the impact of immortal time bias and selection bias, we used target trial emulation, which is a novel rigorous framework specifically designed to address the potential for these study design-related biases.^32^ However, for the estimates to be interpreted in a causal context, some assumptions must be met. Importantly, all patients included should have a non-zero probability of receiving treatment (*positivity*). To meet this assumption, we applied some restrictions to the eligibility criteria. These restrictions were tested in sensitivity analyses. Furthermore, we did not have information on whether chemotherapy was adjuvant or palliative. To increase the probability of only considering adjuvant treatment regimens, we applied an algorithm to detect recurrences before treatment start based on pathology reports from biopsies, ICD codes, and type of chemotherapy administered. However, this algorithm has not been previously validated, and some patients receiving palliative treatment due to very early recurrence or metastatic disease not identified by the algorithm may have been included. We did not have information on whether R2 resections (macroscopically residual tumor) were performed; however, these are rare, with fewer than 10 cases a year in Denmark, and unlikely to have impacted our estimates.^48^ Residual or unmeasured confounding should be eliminated (*exchangeability*). Unmeasured confounding from missing information on performance status^49^ may have contributed to our findings. To mitigate the impact of the missing information on performance status, we included information on other variables that could be associated with both receipt of adjuvant chemotherapy and survival, e.g. comorbidity, ASA score, and albumin.^50^ The postoperative serum albumin recovery rate has been suggested to be a prognostic factor in patients with resected pancreatic adenocarcinoma.^46^ We saw that late initiators had a higher increase in albumin levels than early initiators, as well as higher pretreatment values. This may suggest that late initiators were in better shape when adjuvant chemotherapy was initiated because they had longer time to recover after surgery, although albumin is not a validated marker of postsurgical recovery. An improved performance status would increase the likelihood of patients receiving more efficient combination chemotherapy, although we found no major difference between the two groups according to type of adjuvant chemotherapy.

In our study, a total of 232 (15.6%) patients did not initiate adjuvant chemotherapy. The reason for this is unknown but these patients could potentially have benefited from neoadjuvant therapy. Contrary to adjuvant therapy, neoadjuvant therapy ensures early systemic delivery of chemotherapy and is not contingent on sufficient postoperative recovery. However, neoadjuvant chemotherapy introduces a delay in surgery which, if treatment is inefficient, may render the tumor unresectable. Overall, the use of neoadjuvant versus adjuvant chemotherapy in patients with resectable pancreatic adenocarcinoma is still controversial.^51^

## Conclusion

Our study supports that initiation of adjuvant chemotherapy in pancreatic adenocarcinoma patients can be safely postponed to up to 8 weeks after discharge from surgery, allowing for better recovery after surgery.

### Supplementary Information

Below is the link to the electronic supplementary material.

Supplementary file1 (DOCX 2768 KB)
