# Supplementary material for: Impact on Survival of Early Versus Late Initiation of Adjuvant Chemotherapy After Pancreatic Adenocarcinoma Surgery: A Target Trial Emulation
Source: Ann Surg Oncol. 2023 Nov 1;31(2):1310–8. doi: 10.1245/s10434-023-14497-x (PMC10761389; doi:10.1245/s10434-023-14497-x)
Supplement: Supplementary file 1 — Supplementary file1 (DOCX 2768 KB) [file 10434_2023_14497_MOESM1_ESM.docx]

**SUPPLEMENTARY MATERIAL**

**Impact on Survival of Early versus Late Initiation of Adjuvant Chemotherapy after Pancreatic Adenocarcinoma Surgery: A Target Trial Emulation**

**Authors:**

Jakob Kirkegård (MD, PhD)^1,2^, Morten Ladekarl (MD, DMSc)^3,4^, Andrea Lund (MD)^1,2^, Frank Viborg Mortensen (MD, DMSc)^1,2^

**Affiliations:**

^1^ Department of Surgery, HPB Section; Aarhus University Hospital; Aarhus; Denmark.

^2^ Department of Clinical Medicine; Aarhus University; Aarhus; Denmark.

^3^ Department of Oncology; Aalborg University Hospital; Aalborg; Denmark.

^4^ Department of Clinical Medicine, Aalborg University; Aalborg; Denmark.

**Corresponding author:**

Jakob Kirkegård (MD, PhD, Associate Professor)

Department of Surgery, HPB Section

Aarhus University Hospital

Palle Juul-Jensens Boulevard 35

DK-8200 Aarhus N

Denmark

E-mail: [kirkegaard@clin.au.dk](mailto:kirkegaard@clin.au.dk) / ORCID: 0000-0002-6994-0637

# Table of contents

[Table of contents 2](#_Toc146127132)

[eTable 1: Detailed descriptions of the data sources used 3](#_Toc146127133)

[eTable 2: Specification of the components of the hypothethical target trial and the emulated trial 5](#_Toc146127134)

[eTable 3: List of codes used to identify treatments and recurrences 6](#_Toc146127135)

[eTable 4: List of comorbidity and prescription codes used 7](#_Toc146127136)

[eTable 5: List of codes and weights in the Nordic Multimorbidity Index 8](#_Toc146127137)

[eTable 6. Treatments and procedures used to identify Clavien-Dindo scores 10](#_Toc146127138)

[eTable 7. Covariates included in the weighting models in the target trial emulation 11](#_Toc146127139)

[eTable 8: Descriptive characteristics according to actual treatment initiation 12](#_Toc146127140)

[eTable 9. Tumor and surgery characteristics according to actual treatment initiation 14](#_Toc146127141)

[eTable 10. Results from sensitivity analyses 15](#_Toc146127142)

[STROBE reporting guidelines 16](#_Toc146127143)

[eFigure 1: Flowchart of the study population 18](#_Toc146127144)

[eFigure 2: Directed acyclic graph used to identify potential confounding variables 19](#_Toc146127145)

[eFigure 3: Calculation of weights 20](#_Toc146127146)

[eFigure 4: Standardized mean differences 21](#_Toc146127147)

[eFigure 5: Survival curves from target trial emulation stratified by N-stage 22](#_Toc146127148)

# eTable 1: Detailed descriptions of the data sources used

| **Register** | **Description** | **Covariates** |
| --- | --- | --- |
| **Danish National Patient Registry** | The Danish National Patient Registry was established in 1977. It contains information on all inpatient hospitalizations at Danish public hospitxals. Outpatient and emergency room visits have been included since 1995. Patients are registered in the Danish National Patient Registry with diagnoses coded according to the *International Classification of Diseases* (ICD) 8^th^ revision (ICD-8) from 1977 through 1993 and ICD 10^th^ revision (ICD-10) thereafter. For malignant diagnoses, this registry also contains information on tumor stage according to the tumor-node-metastasis (TNM) classification. Pancreatic cancers were identified as ICD-10 C25. | - Comorbidities - TNM stage - Histology - Surgeries - Chemotherapy - Recurrences - BMI - Smoking |
| **Danish Cancer Registry** | The Danish Cancer Registry includes information on all cancers diagnosed in Denmark since 1943. The registry contains, among other, information on date of diagnosis and tumor location, histology, TNM-stage. | - TNM stage - Histology |
| **Civil Registration System** | The Civil Registration System, which was established in 1968, is an administrative registry containing data on such variables as CPR-number, birth date, sex, sequential dates of migration, marital status, municipality, vital status, and date of death for every resident in Denmark. The Civil Registration System is updated daily and is virtually complete.  Note: Denmark is divided into 98 municipalities, which can be categorized into 1) remote area municipality, 2) rural municipality, 3) regional municipality, and 4) urban/metropolitan municipality. This classification is based on 14 indicators and includes, among others, population size and density, demography, number of workplaces, and socio-economic position. | - Age - Sex - Vital status - Area of residency - Marital status |
| **Danish Anesthesia Database** | The Danish Anesthesia Database was established in 2004 and covers all individuals undergoing a procedure that includes regional or general anaesthesia. It contains information on, among others, alcohol consumption, tobacco smoking, height, weight, and status of physical health according to the American Society of Anesthesiologists (ASA) score at the time of surgical intervention, in addition to information related to the anesthesia and any complications. | - Smoking - Alcohol - ASA score - Height - Weight |
| **Danish Pathology Register** | The Danish Pathology Register contains information on all tissue examinations and pathology specimens examined at Danish hospitals since 1997. The registered data includes information on pathological TNM-stage, resection margins, tumor differentiation, histology, and other potential predictors of cancer prognosis. | - TNM stage - Histology - Metastases - Recurrences |
| **Danish Prescription Registry** | This registry contains detailed information on all filled prescriptions at Danish pharmacies since 1995 with information on, among others, the Anatomical Therapeutic Chemical Classification code, dose and indication of drug, purchase date. | - Prescriptions |
| **Register of Laboratory Results for Research** | The Register of Laboratory Results for Research contains information on biochemical tests performed at Danish laboratories. Denmark is divided into five regions, each of which began reporting to this registry at different times (since 2013), and historical data have been collected from some of the regions as far back as 2008. Coverage varies by region and test date, and data availability depends on transferal from regional laboratory databases to the national registry. The registry includes information on analysis type, results, and sampling date and time. | - Blood samples |

# eTable 2: Specification of the components of the hypothethical target trial and the emulated trial

| **Study component** | **Target trial** | **Emulated trial** |
| --- | --- | --- |
| **Aim** | To estimate the effect of early (0-4 weeks) versus late (>4-8 weeks) initiation of adjuvant chemotherapy after pancreatic adenocarcinoma surgery on survival | Same as target trial |
| **Design** | Two-parallel arm randomised trial | - |
| **Eligibility** | Patients undergoing radical curative-intent surgery for pancreatic adenocarcinoma during 2008-2022 | Same as target trial |
| **Exclusion** | **a)** Non-adenocarcinoma  **b)** Metastases on postoperative pathology  **c)** Neoadjuvant therapy  **d)** Comorbidity/performance status that would prohibit adjuvant therapy | **a)** Same as target trial  **b)** Same as target trial  **c)** Same as target trial  **d)** Clavien-Dindo score >III, ASA score > III, NMI score >20, bilirubin >50, albumin <20, hgb <5 for men, platelets <100, creatinine >100, CA19-9 >200 |
| **Treatment strategies** | **a)** Adjuvant chemotherapy initiated 0-4 weeks after surgery  **b)** Adjuvant chemotherapy initiated >4-8 weeks after surgery | Same as target trial |
| **Treatment assignment** | Randomized and known at baseline | Not randomized, but assumed to be conditionally randomized based on demographic and clinical confounders |
| **Start of follow-up** | Date of randomization | Date of discharge after surgery |
| **Primary endpoint** | Difference in median overall survival | Same as target trial |
| **Secondary endpoints** | Difference in overall survival at 1, 3, and 5 years | Same as target trial |
| **Censoring** | Loss to follow up, administrative censoring | Same as target trial |
| **Type of outcome** | Time-to-event data | Same as target trial |
| **Adjustments** | Age, sex, year of surgery, NMI score, BMI, marital status, area of residency, albumin, postoperative complications, T-stage, N-stage, ASA score | Same as target trial |
| **Sensitivity analyses** | - | **1)** No restriction of inclusion criteria  **2)** Exclusion of ASA III patients  **3)** Restricting to period 2018-2022  **4)** Stratification by N-status |

# eTable 3: List of codes used to identify treatments and recurrences

| **Treatment/procedure/recurrence** | **Code** |
| --- | --- |
| **Pancreatic surgery** | KJLC10, KJLC20, KJLC30, KJLC40 |
| **Chemotherapy** | BWHA (subcodes listed below) |
| Gemcitabine monotherapy | 1, 114 |
| Gemcitabine incl. combinations | 108, 110, 117, 118, 132, 146, 212, 222, 223, 225, 231, 232, 233, 234, 251, 254, 128, 129, 170, 211, 236, 253, 259 |
| FOLFIRINOX (incl. modifications) | 108, 110, 117, 118, 132, 146, 212, 222, 223, 225, 231, 232, 233, 234, 251, 254  BOHC3 |
| *nab*-Paclitaxel | 202, 203, 204, 205, 206, 207, 235, 238 |
| Unknown/unspecified | 2, 3, 4 |
| **Recurrence/metastasis** | C76-C80 |

# eTable 4: List of comorbidity and prescription codes used

| **Comorbidity** | **ICD-10 code** | **ATC code** |
| --- | --- | --- |
| Stroke or other cerebrovascular | I6 | - |
| Cardiac disease | I09.9A, I11.0, I13.0, I13.2, I20-I25, I42-I43, I48, I50, Z94.1 | N02BA C01DA B01AC24 |
| Hypertension | I10, I12-I13, I15 | C08, C03A, C07, C09 |
| Chronic lung disease | J41-J47, J84-J86, G47.3, Z94.2 | R03AK, R03AL, R03BA, R03AC12, R03AC13, R03AC18, R03AC19, R03CC12, R03BB04, R03BB05, R03BB06, R03BB07 |
| Diabetes | E10-E14 | A10 |
| Chronic liver disease | K70-K74, K79, K71-K74, K76.0, K76.6, I85, B15.0, B16.0, B16.2, B18, B19.0 | - |
| Kidney disease | I12-I13, N00-N08, N11, N14, N18-N19, E10.2, E11.2, E14.2, Z94.0 | - |
| Alcohol-related disease | E24.4, E52.9A, F10, G31.2, G62.1, G72.1, I42.6, I85, I98.2, L27.8A, K29.2, K70, O35.4, P04.3, T50.0A, T51.9, X65, Z35.8M10, Z39.310, Z39.320, Z50.2, Z71.4, Z72.1 | N07BB |
| Smoking-related disease | C34, F17, J40-J44, J47, P04.2, T65.2, Z35.8M18, Z39.318, Z58.7 (not A), Z71.6, Z72.0 (not B) | N07BA |
| Psychiatric disease | F2-F9 | N05A, N06B |

# eTable 5: List of codes and weights in the Nordic Multimorbidity Index

| **Comorbidity** | **Weight** | **ICD-10 code** | **ATC code** |
| --- | --- | --- | --- |
| Secondary malignant neoplasms and malignancy of unspecified site* | 22 | C76-C79, C80 | - |
| Malignant neoplasm of bronchus and lung | 19 | C34 | - |
| Alcoholic liver disease, liver fibrosis, cirrhosis, and failure | 13 | K70, K72, K74, K766-K767 | - |
| Mental and behavioural disorders due to use of alcohol | 12 | F10 | - |
| Decubitus ulcer and pressure area | 11 | L89 | - |
| Anti-dementia drugs | 11 | - | N06D |
| Chronic viral hepatitis | 10 | B18 | - |
| Dementia | 9 | F00-F03, G30 | - |
| Leukemia | 8 | C91-C95 | - |
| Malignant neoplasm of bladder | 8 | C67 | - |
| Drugs for constipation | 8 | - | A06A |
| Tumor of brain or meninges | 8 | C70, C71, C751-C753, D32, D330-D332, D352-D354, D42,  D430-D432, D443-D445 | - |
| Multiple sclerosis | 7 | G35 | - |
| Other interstitial pulmonary diseases | 7 | J84 | - |
| Drugs used in opioid dependence | 7 | - | N07BC |
| Parkinson’s disease and other parkinsonism | 7 | G20-G22 | - |
| Antipsychotics | 7 | - | N05A excl. N05AN |
| Chronic kidney disease and unspecified kidney failure | 7 | N18-N19 | - |
| Volume depletion | 6 | E86 | - |
| Atherosclerosis, thrombosis, embolism, and other peripheral arterial disease | 5 | I70, I73-I74, I77 | - |
| Iron preparations | 5 | - | B03A |
| Antipropulsives | 5 | - | A07DA |
| Diseases of teeth and supporting structures | 5 | K02-K06, K08 | - |
| High-ceiling diuretics | 5 | - | C03C, C03EB |
| Long-acting anti-muscarinic agents | 5 | - | R03BB04-07 |
| Anaemia | 5 | D50-D59, D60-D64 | - |
| Malignant neoplasm of prostate | 5 | C61 | - |
| Epilepsy | 5 | G40-G41 | - |
| Insulins and analogues | 4 | - | A10A |
| Pneumonia | 4 | J12-J18 | - |
| Chronic lower respiratory diseases and failure | 4 | J41-J44, J47, J961, J969 | - |
| Digitalis glycosides | 4 | - | C01AA |
| Malignant neoplasm of breast | 4 | C50 | - |
| Cerebrovascular disease | 4 | I60-I69 | - |
| Aneurysm and dissection of aorta and other arteries | 4 | I71-I72 | - |
| Mental and behavioural disorders due to use of tobacco | 4 | F17 | - |
| Heart failure | 4 | I110, I130, I132, I420, I426-I429, I50 | - |
| Short-acting beta agonists | 3 | - | R03AC02-05 |
| Aldosterone antagonists | 3 | - | C03DA |
| Antidepressants | 3 | - | N06A |
| Opioids | 2 | - | N02A |
| Anilides | 2 | - | N02BE |
| Type 2 diabetes mellitus | 2 | E11 | - |
| Aortic and mitral valve disease | 2 | I05-I06, I34-I35 | - |
| Glucocorticoids for systemic use | 2 | - | H02AB |
| Platelet aggregation inhibitors excl. heparin | 2 | - | B01AC |
| Benzodiazepines and related drugs | 1 | - | N05BA, N05CD, N05CF |
| Beta-lactam antibacterials, penicillins | 1 | - | J01C |
| ARBs incl. combinations | -2 | - | C09C, C09D |
| HMG CoA reductase inhibitors (statins) | -3 | - | C10AA |

* Excluded from calculations.

# eTable 6. Treatments and procedures used to identify Clavien-Dindo scores

| **Clavien-Dindo** | **Complication score** | **Code (including sublevels)** |
| --- | --- | --- |
| **Clavien-Dindo 0-II** | **Low** | **ZDA03A0, ZDA03A1, ZDA03A2** |
| None of the codes listed below |  |  |
| **Clavien-Dindo III** | **High** | **ZDA03A3** |
| Upper or lower endoscopy |  | KUJC, KUJD, KUJF |
| ERCP |  | KUJK |
| Percutaneous drainge of the abdomen |  | BIBG |
| Operative pleural drainage |  | KGAA |
| Any abdominal reoperation |  | KJ |
| Negative wound pressure therapy |  | BNPA92 |
| **Clavien-Dindo IV** | ***(Excluded)*** | **ZDA03A4** |
| Mechanical ventilation |  | BGDA |
| ECMO |  | BGXA2, KFXE, ZZP0004F |
| Acute dialysis |  | BJFD |
| Use of inotropy |  | BFHC92 |

# eTable 7. Covariates included in the weighting models in the target trial emulation

| **Covariate** | **Type** | **Coding** |
| --- | --- | --- |
| Age | Continuous | Restricted cubic spline (4 knots) |
| Sex | Categorical | 1: Men  2: Women |
| Year of diagnosis | Continuous | Restricted cubic spline (3 knots) |
| Area of residence | Categorical | 0: Remote, rural, or regional municipality  1: Urban/metropolitan municipality |
| Marital status | Categorical | 0: Unmarried/divorced/widowed  1: Married |
| NMI score^a^ | Continuous | Restricted cubic spline (3 knots) |
| ASA score^a^ | Categorical | 1: ASA I  2: ASA II  3: ASA III |
| BMI^a^ | Continuous | Restricted cubic spline (3 knots) |
| Albumin level^a^ | Continuous | Restricted cubic spline (3 knots) |
| T-stage | Categorical | 1: T1  2: T2  3: T3  4: T4 |
| N-stage | Categorical | 0: N-  1: N+ |
| Complication score | Categorical | 0: Low  1: High |

^a^ Updated daily througout the grace period as time-varying covariates.

# eTable 8: Descriptive characteristics according to actual treatment initiation

|  | | **Never** | **0-4 weeks** | **>4-8 weeks** | **>8 weeks** |
| --- | --- | --- | --- | --- | --- |
| **Total** | | 232 (15.6%) | 482 (32.3%) | 571 (38.3%) | 206 (13.8%) |
| **Type of adjuvant chemotherapy** | |  |  |  |  |
|  | Gemcitabine monotherapy | - | 293 (60.8%) | 343 (60.1%) | - |
|  | Gemcitabine combination | - | 101 (21.0%) | 128 (22.4%) | - |
|  | FOLFIRINOX incl. modifications | - | 88 (18.3%) | 100 (17.5%) | - |
|  | Unknown | 232 (100%) | - | - | 206 (100%) |
| **Time to initiation, *days*, median (IQR)** | | - | 23 (19-26) | 36 (33-43) | - |
| **Age, *years*, median (IQR)** | | 73 (66-78) | 67 (60-72) | 68 (62-73) | 68 (63-74) |
| **Age group** | |  |  |  |  |
|  | <60 years | 22 (9.5%) | 129 (26.8%) | 127 (22.2%) | 40 (19.4%) |
|  | 61-70 years | 62 (26.7%) | 185 (38.4%) | 230 (40.3%) | 80 (38.8%) |
|  | 71-80 years | 148 (63.8%) | 168 (34.9%) | 214 (37.5%) | 86 (41.7%) |
| **Sex** | |  |  |  |  |
|  | Men | 114 (49.1%) | 258 (53.5%) | 291 (51.0%) | 108 (52.4%) |
|  | Women | 118 (50.9%) | 224 (46.5%) | 280 (49.0%) | 98 (47.6%) |
| **Marital status** | |  |  |  |  |
|  | Married/registered partner | 105 (45.3%) | 221 (45.9%) | 266 (46.6%) | 92 (44.7%) |
|  | Unmarried/divorced/widowed | 127 (54.7%) | 261 (54.1%) | 305 (53.4%) | 114 (55.3%) |
| **Area of residence** | |  |  |  |  |
|  | Urban municipality | 130 (56.0%) | 351 (72.8%) | 363 (63.6%) | 138 (67.0%) |
|  | Rural municipality | 102 (44.0%) | 131 (27.2%) | 208 (36.4%) | 68 (33.0%) |
| **Calendar period of diagnosis** | |  |  |  |  |
|  | 2008-2014 | 62 (26.7%) | 169 (35.1%) | 152 (26.6%) | 63 (30.6%) |
|  | 2015-2018 | 70 (30.2%) | 156 (32.4%) | 178 (31.2%) | 73 (35.4%) |
|  | 2019-2022 | 100 (43.1%) | 157 (32.6%) | 241 (42.2%) | 70 (34.0%) |
| **Alcohol consumption** | |  |  |  |  |
|  | None | 22 (9.5%) | 45 (9.3%) | 70 (12.3%) | 23 (11.2%) |
|  | 1-14 units/week | 28 (12.1%) | 95 (19.7%) | 89 (15.6%) | 29 (14.1%) |
|  | 15-21 units/week | n<=5 | n<=5 | n<=5 | n<=5 |
|  | >21 units/week | n<=5 | 6 (1.2%) | 6 (1.1%) | n<=5 |
|  | Unknown | 179 (77.2%) | n>320 | n>400 | 152 (73.8%) |
| **Tobacco smoking** | |  |  |  |  |
|  | Non-smoker | 59 (25.4%) | 120 (24.9%) | 145 (25.4%) | 39 (18.9%) |
|  | Current smoker | 16 (6.9%) | 40 (8.3%) | 62 (10.9%) | 25 (12.1%) |
|  | Previous smoker | n<=5 | 29 (6.0%) | 12 (2.1%) | n>5 |
|  | Unknown | n>150 | 293 (60.8%) | 352 (61.6%) | n>125 |
| **Nordic Multimorbidity Index, mean (SD)** | | 3.9 (4.8) | 2.8 (4.1) | 3.1 (4.6) | 3.4 (5.0) |
| **Body Mass Index, mean (SD)** | | 24.5 (3.1) | 24.9 (3.1) | 24.7 (3.1) | 25.0 (3.1) |
| **ASA score** | |  |  |  |  |
|  | ASA I | n<=5 | 9 (1.9%) | 8 (1.4%) | n<=5 |
|  | ASA II | 112 (48.3%) | 315 (65.4%) | 342 (59.9%) | 119 (57.8%) |
|  | ASA III | n>115 | 158 (32.8%) | 221 (38.7%) | n>80 |
| **Comorbidity** | |  |  |  |  |
|  | Stroke or other cerebrovascular | 7 (3.0%) | 8 (1.7%) | 15 (2.6%) | 10 (4.9%) |
|  | Cardiac disease | 56 (24.1%) | 63 (13.1%) | 95 (16.6%) | 44 (21.4%) |
|  | Hypertension | 130 (56.0%) | 250 (51.9%) | 313 (54.8%) | 114 (55.3%) |
|  | Chronic lung disease | 47 (20.3%) | 81 (16.8%) | 84 (14.7%) | 31 (15.0%) |
|  | Diabetes | 90 (38.8%) | 128 (26.6%) | 165 (28.9%) | 61 (29.6%) |
|  | Chronic liver disease | n<=5 | 7 (1.5%) | 8 (1.4%) | n<=5 |
|  | Kidney disease | n<=5 | 6 (1.2%) | n<=5 | n<=5 |
|  | Alcohol-related disease | 11 (4.7%) | 10 (2.1%) | 23 (4.0%) | 6 (2.9%) |
|  | Smoking-related disease | 30 (12.9%) | 55 (11.4%) | 73 (12.8%) | 30 (14.6%) |
|  | Psychiatric disease | 14 (6.0%) | 13 (2.7%) | 33 (5.8%) | 14 (6.8%) |
| **Blood tests** | |  |  |  |  |
|  | CRP, mg/L | 28 (14-67) | 26 (12-53) | 31 (15-61) | 33 (14-65) |
|  | Hgb mmol/L | 6 (6-7) | 6 (6-7) | 6 (6-7) | 6 (6-6) |
|  | Platelets, 10^9^/L | 10 (7-13) | 10 (8-12) | 10 (8-12) | 10 (8-12) |
|  | Leukocytes, 10^9^/L | 414 (310-540) | 404 (290-496) | 384 (261-500) | 373 (253-496) |
|  | Albumin, g/L | 27 (25-29) | 27 (25-30) | 27 (26-30) | 27 (25-29) |
|  | Bilirubin, umol/L | 10 (6-19) | 14 (8-22) | 12 (7-21) | 11 (7-22) |
|  | CA19-9, U/L | 80 (80-80) | 46 (31-118) | 57 (20-99) | 80 (26-135) |
|  | Creatinine, umol/L | 58 (51-70) | 60 (50-69) | 58 (49-68) | 57 (47-69) |

Numbers masked in some cells due to protection of individual-level data according to Danish law.

# eTable 9. Tumor and surgery characteristics according to actual treatment initiation

|  | | **Never** | **0-4 weeks** | **>4-8 weeks** | **>8 weeks** |
| --- | --- | --- | --- | --- | --- |
| **Total** | | 232 (15.6%) | 482 (32.3%) | 571 (38.3%) | 206 (13.8%) |
| **Tumor location** | |  |  |  |  |
|  | Head | 142 (61.2%) | 321 (66.6%) | 363 (63.6%) | 120 (58.3%) |
|  | Body | 10 (4.3%) | 39 (8.1%) | 39 (6.8%) | 16 (7.8%) |
|  | Tail | 21 (9.1%) | 19 (3.9%) | 52 (9.1%) | 17 (8.3%) |
|  | Multiple | 15 (6.5%) | 24 (5.0%) | 36 (6.3%) | 16 (7.8%) |
|  | Unknown | 44 (19.0%) | 79 (16.4%) | 81 (14.2%) | 37 (18.0%) |
| **Tumor size, *mm*, median (IQR)** | | 27 (19-35) | 30 (23-36) | 30 (24-40) | 28 (21-37) |
| **T-stage** | |  |  |  |  |
|  | T1 | 29 (12.5%) | 22 (4.6%) | 59 (10.3%) | 16 (7.8%) |
|  | T2 | 86 (37.1%) | 134 (27.8%) | 179 (31.3%) | 62 (30.1%) |
|  | T3 | 109 (47.0%) | 311 (64.5%) | 317 (55.5%) | 114 (55.3%) |
|  | T4 | 8 (3.4%) | 15 (3.1%) | 16 (2.8%) | 14 (6.8%) |
| **N-stage** | |  |  |  |  |
|  | N0 | 102 (44.0%) | 126 (26.1%) | 144 (25.2%) | 70 (34.0%) |
|  | N+ | 130 (56.0%) | 356 (73.9%) | 427 (74.8%) | 136 (66.0%) |
| **AJCC stage** | |  |  |  |  |
|  | Stage I | 65 (28.0%) | 52 (10.8%) | 70 (12.3%) | 38 (18.4%) |
|  | Stage II | 138 (59.5%) | 346 (71.8%) | 380 (66.5%) | 122 (59.2%) |
|  | Stage III | 29 (12.5%) | 84 (17.4%) | 121 (21.2%) | 46 (22.3%) |
| **Tumor differentiation** | |  |  |  |  |
|  | Poor | 25 (10.8%) | 42 (8.7%) | 70 (12.3%) | 25 (12.1%) |
|  | Moderate | 34 (14.7%) | 88 (18.3%) | 107 (18.7%) | 40 (19.4%) |
|  | High | n>5 | 12 (2.5%) | 22 (3.9%) | n<=5 |
|  | Unknown | n>160 | 340 (70.5%) | 372 (65.1%) | n>130 |
| **Neural invasion** | |  |  |  |  |
|  | No | 14 (6.0%) | 15 (3.1%) | 18 (3.2%) | 6 (2.9%) |
|  | Yes | 53 (22.8%) | 116 (24.1%) | 183 (32.0%) | 53 (25.7%) |
|  | Unknown | 165 (71.1%) | 351 (72.8%) | 370 (64.8%) | 147 (71.4%) |
| **Vascular invasion** | |  |  |  |  |
|  | No | 25 (10.8%) | 34 (7.1%) | 55 (9.6%) | 17 (8.3%) |
|  | Yes | 46 (19.8%) | 105 (21.8%) | 137 (24.0%) | 39 (18.9%) |
|  | Unknown | 161 (69.4%) | 343 (71.2%) | 379 (66.4%) | 150 (72.8%) |
| **Microradical resection** | |  |  |  |  |
|  | No | 127 (54.7%) | 241 (50.0%) | 328 (57.4%) | 106 (51.5%) |
|  | Yes | 12 (5.2%) | 53 (11.0%) | 44 (7.7%) | 27 (13.1%) |
|  | Unknown | 93 (40.1%) | 188 (39.0%) | 199 (34.9%) | 73 (35.4%) |
| **Type of surgery** | |  |  |  |  |
|  | Pancreatoduodenectomy | 26 (11.2%) | 46 (9.5%) | 84 (14.7%) | 30 (14.6%) |
|  | Distal pancreatectomy | 181 (78.0%) | 380 (78.8%) | 428 (75.0%) | 157 (76.2%) |
|  | Total pancreatectomy | 25 (10.8%) | 56 (11.6%) | 59 (10.3%) | 19 (9.2%) |
| **Length of stay, *days*, median (IQR)** | | 11 (8-16) | 11 (8-15) | 9 (7-13) | 10 (8-15) |
| **Complication score** | |  |  |  |  |
|  | Low (Clavien-Dindo 0-II) | 193 (83.2%) | 410 (85.1%) | 492 (86.2%) | 163 (79.1%) |
|  | High (Clavien-Dindo III) | 39 (16.8%) | 72 (14.9%) | 79 (13.8%) | 43 (20.9%) |

# eTable 10. Results from sensitivity analyses

|  | | **Survival, % (95% CI)** | | | **mOS (IQR) (95% CI for difference)** |
| --- | --- | --- | --- | --- | --- |
|  | | **1 year** | **3 years** | **5 years** |  |
| **Analysis 1** | |  |  |  |  |
|  | Late | 80.7 (77.5; 83.8) | 42.9 (38.8; 47.0) | 29 (25.1; 32.9) | 29.4 (14.7; NR) |
|  | Early | 76.3 (72.7; 79.9) | 43.5 (39.1; 47.9) | 26.6 (22.4; 30.7) | 28.9 (12.8; NR) |
|  | Difference | 4.4 (0.1; 8.7) | -0.6 (-6.4; 5.3) | 2.4 (-2.8; 7.7) | 0.5 (-4.6; 5.6) |
| **Analysis 2** | |  |  |  |  |
|  | Late | 83.4 (79.7; 87.1) | 45.5 (39.9; 51.0) | 34.1 (28.3; 39.8) | 30.9 (16.5; NR) |
|  | Early | 83.4 (79.1; 87.6) | 47.8 (42.0; 53.6) | 29.1 (23.3; 34.9) | 31.8 (15.8; NR) |
|  | Difference | 0.0 (-5.4; 5.4) | -2.3 (-10.1; 5.4) | 5.0 (-3.0; 12.9) | -0.8 (-8.5; 6.9) |

1: Less restrictive approach to definition of operability. No restrictions on blood samples, complications, or comorbidity. N=1,744.

2: Exclusion of ASA III patients. N=910.

NR: not reached

# STROBE reporting guidelines

|  | | Item | | Recommendation | Page |
| --- | --- | --- | --- | --- | --- |
| **Title and abstract** | | 1 | | (*a*) Indicate the study’s design with a commonly used term in the title or the abstract | 1 |
|  |  |  |  | (*b*) Provide in the abstract an informative and balanced summary of what was done and what was found | 3 |
| Introduction | | | | | |
| Background/rationale | | 2 | | Explain the scientific background and rationale for the investigation being reported | 5 |
| Objectives | | 3 | | State specific objectives, including any prespecified hypotheses | 5 |
| Methods | | | | | |
| Study design | | 4 | | Present key elements of study design early in the paper | 6 |
| Setting | | 5 | | Describe the setting, locations, and relevant dates, including periods of recruitment, exposure, follow; up, and data collection | 6 |
| Participants | | 6 | | (*a*) Give the eligibility criteria, and the sources and methods of selection of participants. Describe methods of follow; up | 6-7, eFigure 1, eTable 2 |
|  |  |  |  | (*b*) For matched studies, give matching criteria and number of exposed and unexposed | - |
| Variables | | 7 | | Clearly define all outcomes, exposures, predictors, potential confounders, and effect modifiers. Give diagnostic criteria, if applicable | 7-8 |
| Data sources/ measurement | | 8* | | For each variable of interest, give sources of data and details of methods of assessment (measurement). Describe comparability of assessment methods if there is more than one group | eTable 1 |
| Bias | | 9 | | Describe any efforts to address potential sources of bias | 8, eFigure 3 |
| Study size | | 10 | | Explain how the study size was arrived at | eFigure 1 |
| Quantitative variables | | 11 | | Explain how quantitative variables were handled in the analyses. If applicable, describe which groupings were chosen and why | eTable 7 |
| Statistical methods | | 12 | | (*a*) Describe all statistical methods, including those used to control for confounding | 8-10 |
|  |  |  |  | (*b*) Describe any methods used to examine subgroups and interactions | 10 |
|  |  |  |  | (*c*) Explain how missing data were addressed | 8 |
|  |  |  |  | (*d*) If applicable, explain how loss to follow; up was addressed | - |
|  |  |  |  | (*e*) Describe any sensitivity analyses | 10 |
| Results | | | | |  |
| Participants | | 13* | | (a) Report numbers of individuals at each stage of study—eg numbers potentially eligible, examined for eligibility, confirmed eligible, included in the study, completing follow; up, and analysed | 10, eFigure 1 |
|  |  |  |  | (b) Give reasons for non; participation at each stage | eFigure 1 |
|  |  |  |  | (c) Consider use of a flow diagram | eFigure 1 |
| Descriptive data | | 14* | | (a) Give characteristics of study participants (eg demographic, clinical, social) and information on exposures and potential confounders | 10, Table 1-2 |
|  |  |  |  | (b) Indicate number of participants with missing data for each variable of interest | Table 1 |
|  |  |  |  | (c) Summarise follow; up time (eg, average and total amount) | 11 |
| Outcome data | | 15* | | Report numbers of outcome events or summary measures over time | 11 |
| Main results | | 16 | | (a) Give unadjusted estimates and, if applicable, confounder; adjusted estimates and their precision (eg, 95% confidence interval). Make clear which confounders were adjusted for and why they were included | 11, Table 3 |
|  | |  | | (b) Report category boundaries when continuous variables were categorized | - |
|  | |  | | (c) If relevant, consider translating estimates of relative risk into absolute risk for a meaningful time period | Table 3 |
| Other analyses | | 17 | | Report other analyses done—eg analyses of subgroups and interactions, and sensitivity analyses | 11-12 |
| Discussion | | | | | |
| Key results | 18 | | Summarise key results with reference to study objectives | | 12 |
| Limitations | 19 | | Discuss limitations of the study, taking into account sources of potential bias or imprecision. Discuss both direction and magnitude of any potential bias | | 13-14 |
| Interpretation | 20 | | Give a cautious overall interpretation of results considering objectives, limitations, multiplicity of analyses, results from similar studies, and other relevant evidence | | 13-14 |
| Generalisability | 21 | | Discuss the generalisability (external validity) of the study results | | 13-14 |
| Other information | | | | | |
| Funding | 22 | | Give the source of funding and the role of the funders for the present study and, if applicable, for the original study on which the present article is based | | 1-2 |

# eFigure 1: Flowchart of the study population

**
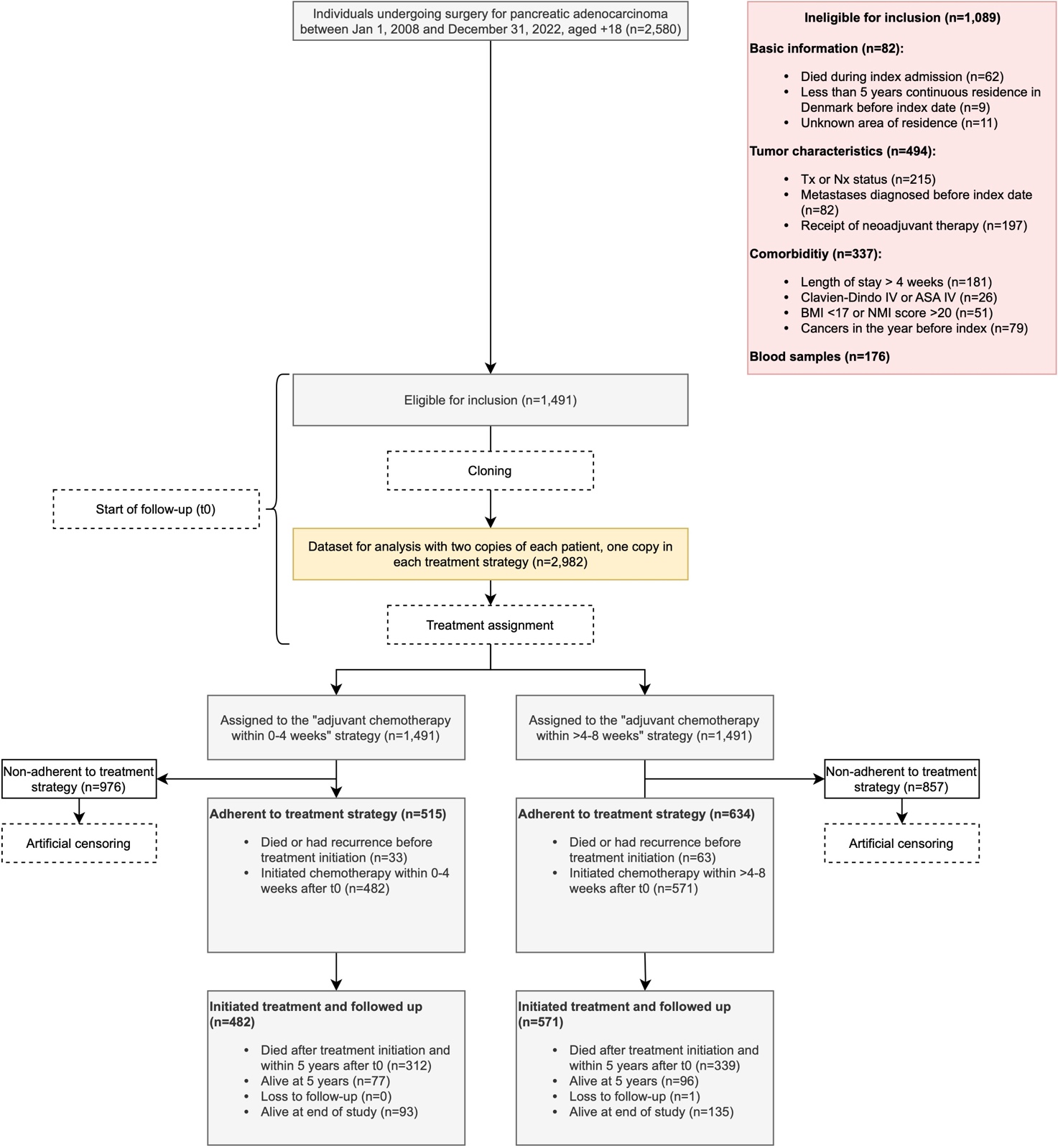
**

# eFigure 2: Directed acyclic graph used to identify potential confounding variables


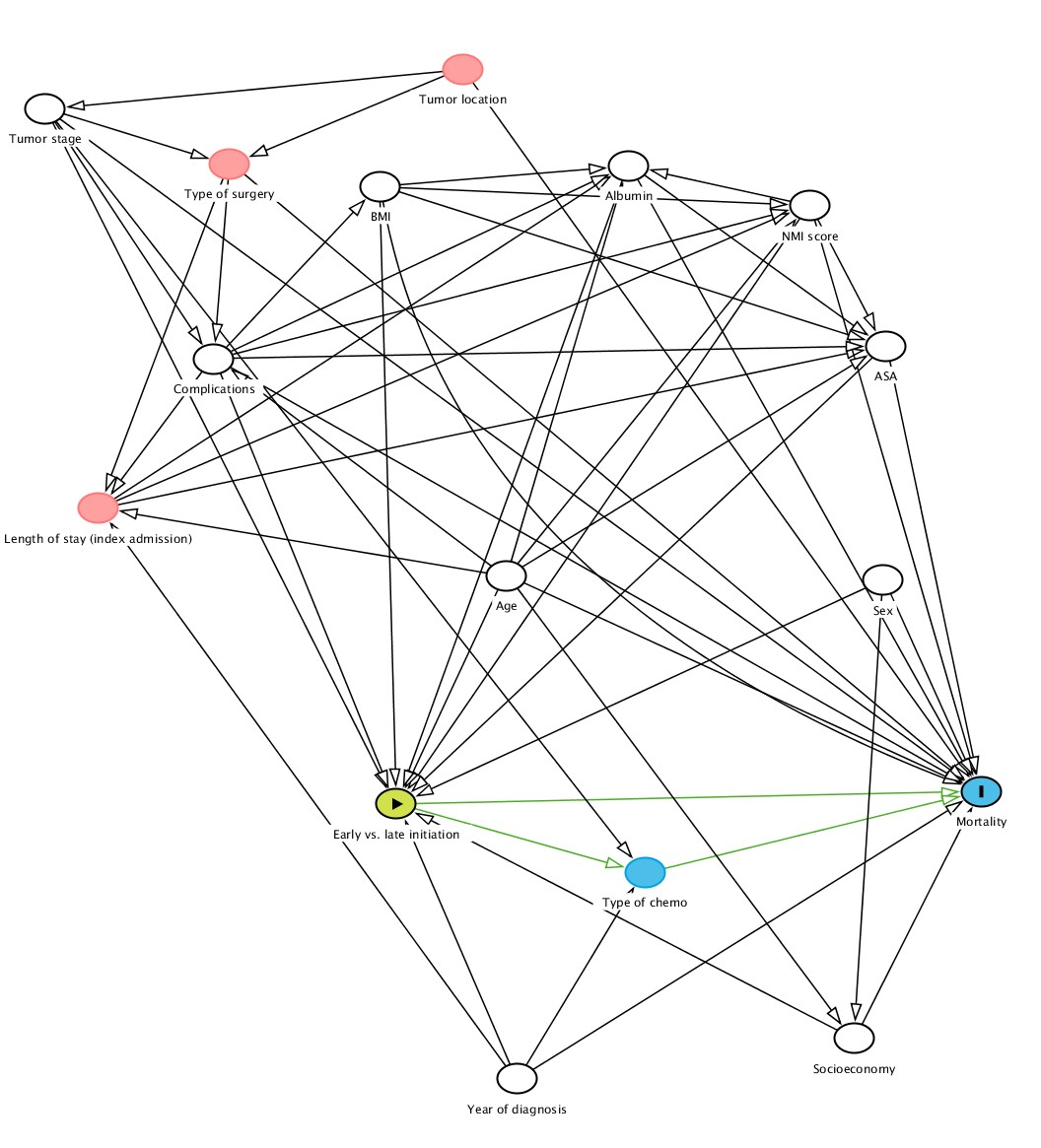


Minimal sufficient set of confounders: ASA, Age, Albumin, BMI, CA19-9, Complications, NMI score, Sex, Socioeconomy, Tumor stage, Year of diagnosis. Socioeconomy is approximated using “marital status” and “area of residence”.

# eFigure 3: Calculation of weights

TIME: 0 DAYS TIME: 28 DAYS TIME: 56 DAYS

Early 1 Pr(uncensored)/Pr(uncensored|covariates)

**R**

Late Pr(uncensored)/Pr(uncensored|covariates) 1 Pr(uncensored)/Pr(uncensored|covariates)

In the early initiation strategy, patients could only deviate after 4 weeks. In the late initiation strategy, patients could deviate at any time during the first 0-4 weeks and again at the end of the 8-week grace period. Treatment-specific weights were calculated using a pooled logistic regression model. In the early initiation strategy, weights were set to 1 during day 0-27 and then calculated at day 28. In the late initiation strategy, we included day of follow-up since the index date as a predictor, modelled as a restricted cubic spline with four knots. Weights were calculated daily on days 0-28 and again at day 56. At day 29-55, weights were set to 1.

*R: "Randomization"*

# eFigure 4: Standardized mean differences


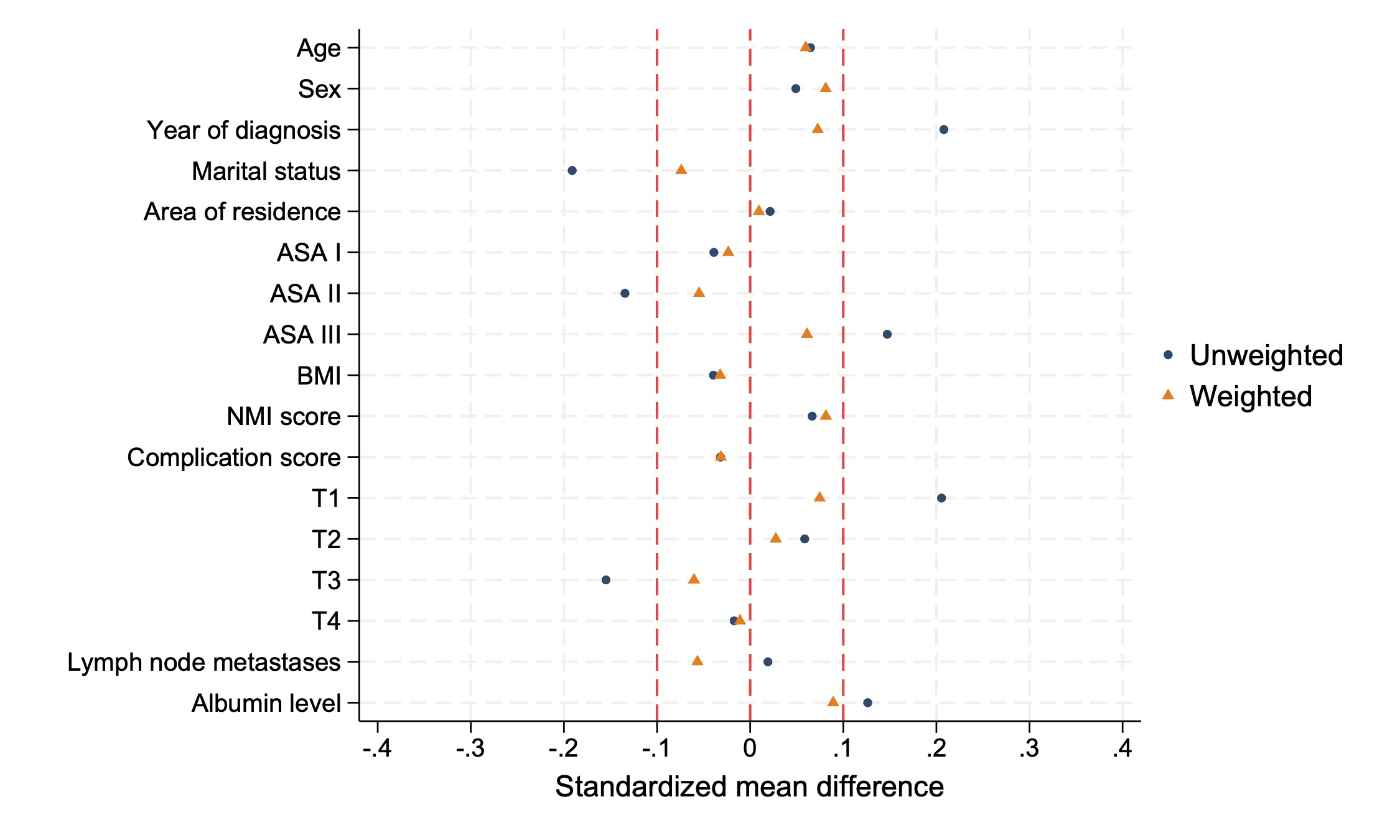


Standardized mean differences at the end of the 8-week grace period, comparing the early and late initiation strategies.

# eFigure 5: Survival curves from target trial emulation stratified by N-stage

| **A: N+ disease** | **B: N0 disease** |
| --- | --- |
| 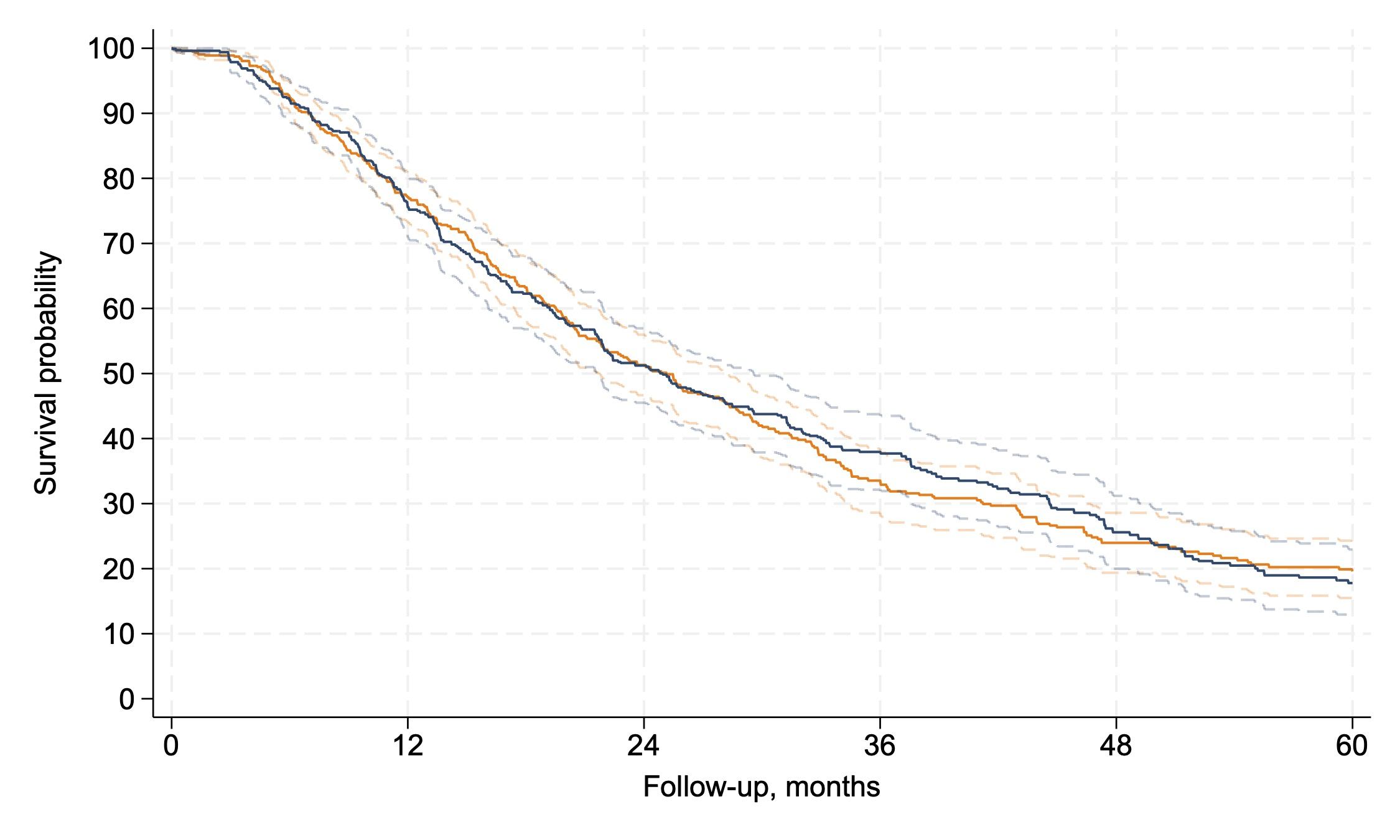 | 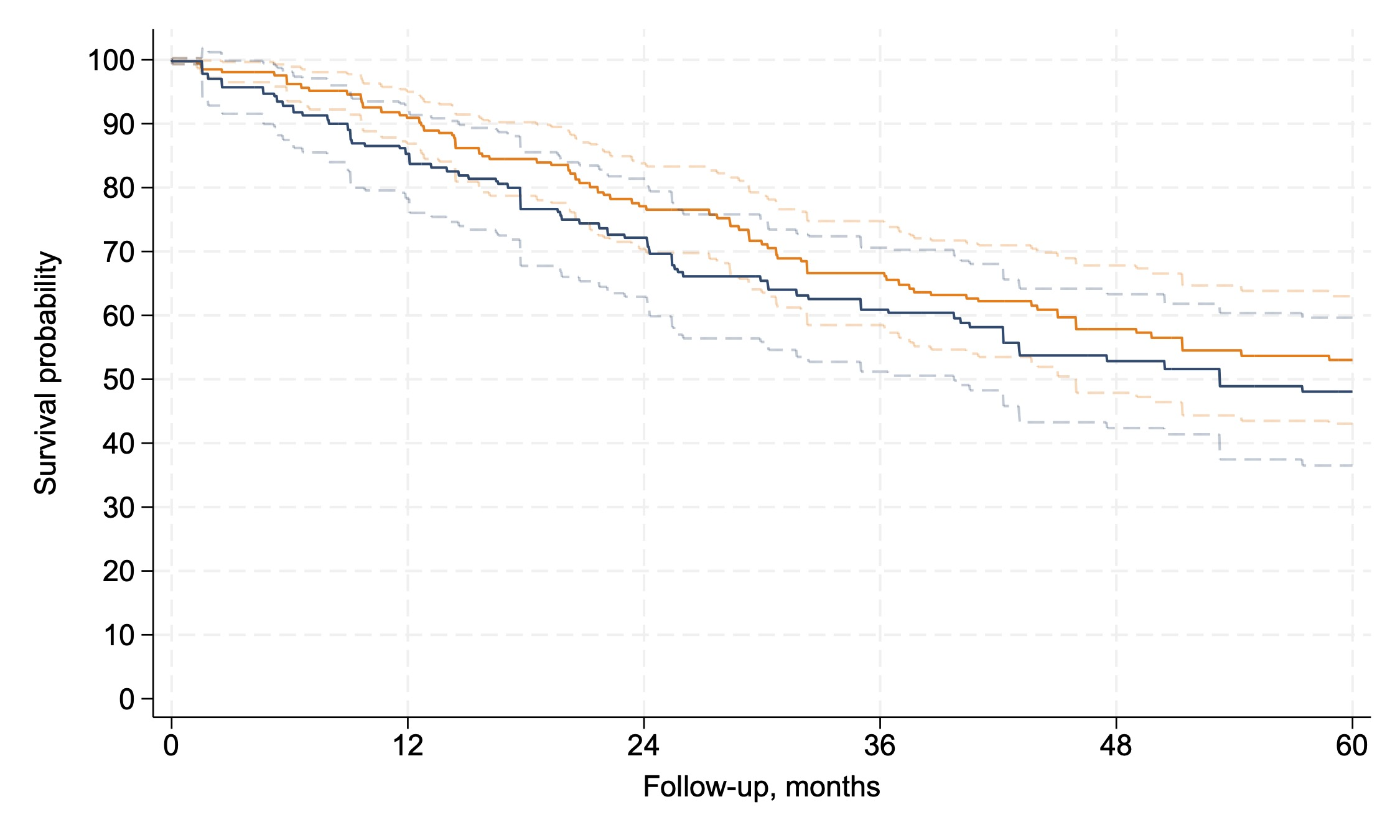 |

Orange: Initiation >4-8 weeks; blue: initiation 0-4 weeks.
